# Ethyl 3-benzoyl­indolizine-1-carboxyl­ate

**DOI:** 10.1107/S160053681104284X

**Published:** 2011-10-29

**Authors:** Wei-Jin Gu, Jin Zhuang, Yu-Liang Jiang, Bing-Xiang Wang

**Affiliations:** a Department of Applied Chemistry, Jiangsu Key Laboratory of Biofunctional Materials, Jiangsu Research Center of Biomedical Functional Materials Engineering, Nanjing Normal University, Nanjing 210097, People’s Republic of China

## Abstract

The title compound, C_18_H_15_NO_3_, consists of an indolizine ring system and an aromatic ring. The two ring systems are not coplanar, the dihedral angle between the two being 54.26 (7)°. In the crystal, inversion dimers are formed by weak C—H⋯O interactions. These dimeric groups are further extended to form a regular two-dimensional structure by additional weak C—H⋯O inter­actions.

## Related literature

For background information on indolizine and its derivatives, see: Tukulula *et al.* (2010 ▶); James *et al.* (2008 ▶); Teklu *et al.* (2005 ▶); Shen *et al.* (2008 ▶, 2006 ▶). For the synthesis of the title compound, see: Wang *et al.* (2000 ▶).
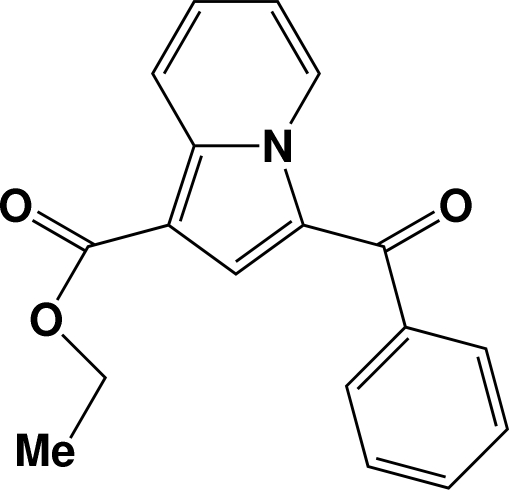


## Experimental

### 

#### Crystal data

C_18_H_15_NO_3_
*M* *_r_* = 293.31
Monoclinic, 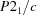

*a* = 10.030 (2) Å
*b* = 19.223 (3) Å
*c* = 7.9652 (17) Å
β = 103.073 (3)°
*V* = 1495.9 (5) Å^3^
*Z* = 4
Mo *K*α radiation
μ = 0.09 mm^−1^
*T* = 291 K
0.24 × 0.20 × 0.18 mm

#### Data collection

Bruker SMART APEX CCD diffractometer
Absorption correction: multi-scan (*SADABS*; Bruker, 2000 ▶) *T*_min_ = 0.979, *T*_max_ = 0.984
8910 measured reflections
2604 independent reflections
1604 reflections with *I* > 2σ(*I*)
*R*_int_ = 0.057

#### Refinement

*R*[*F* ^2^ > 2σ(*F* ^2^)] = 0.048
*wR*(*F* ^2^) = 0.129
*S* = 1.00
2604 reflections
201 parameters
H-atom parameters constrained
Δρ_max_ = 0.18 e Å^−3^
Δρ_min_ = −0.15 e Å^−3^

### 

Data collection: *SMART* (Bruker, 2000 ▶); cell refinement: *SAINT* (Bruker, 2000 ▶); data reduction: *SAINT*; program(s) used to solve structure: *SHELXTL* (Sheldrick, 2008 ▶); program(s) used to refine structure: *SHELXTL*; molecular graphics: *SHELXTL*; software used to prepare material for publication: *SHELXTL*.

## Supplementary Material

Crystal structure: contains datablock(s) global, I. DOI: 10.1107/S160053681104284X/im2326sup1.cif

Structure factors: contains datablock(s) I. DOI: 10.1107/S160053681104284X/im2326Isup2.hkl

Supplementary material file. DOI: 10.1107/S160053681104284X/im2326Isup3.cml

Additional supplementary materials:  crystallographic information; 3D view; checkCIF report

## Figures and Tables

**Table 1:** Hydrogen-bond geometry (Å, °)

*D*—H⋯*A*	*D*—H	H⋯*A*	*D*⋯*A*	*D*—H⋯*A*
C1—H1⋯O1^i^	0.93	2.45	3.163 (3)	134
C14—H14⋯O3^ii^	0.93	2.58	3.455 (3)	157

Symmetry codes: (i) 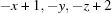; (ii) 
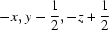
.

**Fig. 1.**
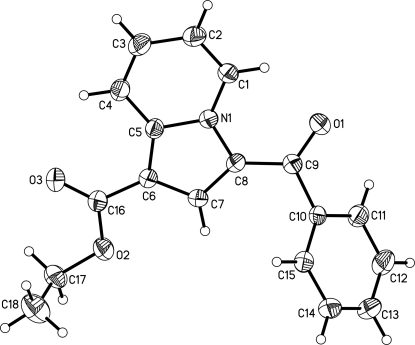
A view of the title compound showing the atom-numbering scheme and displacement ellipsoids drawn at 30% probability level.

**Fig. 2.**
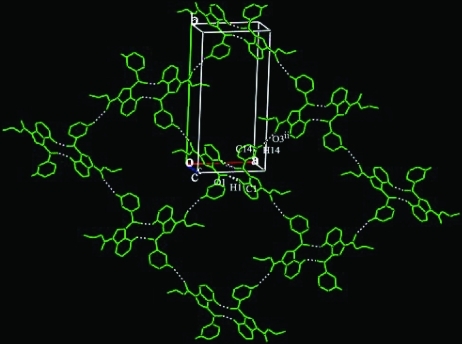
A view of the 2-D structure down c axis (i: 1 - x,-y,2 - z; ii: -x,-1/2 + y,0.5 - z).

## supplementary crystallographic information

### Comment

Indolizine and it's derivatives have been comprehensively applied in biology and medicine due to their particular structures (Tukulula *et al.*, 2010; James *et al.*, 2008; Teklu *et al.*, 2005). They can also be used as organic fluorescence probes (Shen *et al.*, 2008; Shen *et al.*, 2006). In our continuing studies in organic fluorescence probes, we synthesized the ethyl-3-benzoylindolizine-1-carboxylate (I).

The crystal structure of the title compound, C_18_H_15_NO_3_, reveals that all bond lengths and angles have normal values (Table 1 and 2). In the asymmetric unit there is one title compound molecule. The molecular structure consists of one indolizine ring A (C1—C8/N) and an aromatic ring B(C10—C15) (Fig. 1). Rings A and ring B are not coplanar with the dihedral angle between them being 54.26 (7) °.

In the crystal packing there are weak C1—H1···O1^i^ (i: 1 - *x*,-*y*,2 - *z*) interactions between neighbouring molecules forming dimeric groups (Fig. 2). These dimeric groups are further extended into a tegular 2-D structure *via* weak C14—H14···O3^ii^ (ii: -*x*,-1/2 + *y*,0.5 - *z*) interactions (Fig. 2).

### Experimental

Ethyl-3-benzoylindolizine-1-carboxylate was prepared by 1,3-dipolar cycloaddition according to a procedure described in the literature (Wang, *et al.*, 2000). A suspension of *N*-(benzoylmethyl)pyridinium bromide (C_5_H_5_N^+^–CH_2_COC_6_H_5_ Br^-^) (10 mmol), ethyl acrylate (40 mmol), Et_3_N (20 ml) and CrO_3 _(20 mmol) in DMF (40 ml) was stirred at 90°C for 2 h (monitored by TLC). The mixture was then cooled to room temperature and poured into 5% aqueous HCl (200 mL). The mixture was extracted with CH_2_Cl_2_ (2 times 50 mL) and the combined extracts were washed with water (2 times 50 mL) and dried over Na_2_SO_4_. The solvent was removed to give a solid, which was purified by chromatography [silica gel, 20% ethyl acetate in light petroleum (b.p. 60–90°C)] to yield 1.90 g (68%) (I). Yellow crystals were obtained by recrystallization from ethyl acetate at room temperature.

H-NMR (CDCl_3_, 400 MHz) δ: 1.41 (t, 3H, CH_3_), 4.38 (q, 2H, CH_2_), 7.10 (t, 1H, H6), 7.44–7.84 (m, 7H, H7, H2 and PhH), 8.39 (d, 1H, H8), 9.98 (d, 1H, H5).

### Refinement

The H atoms were placed in calculated positions and included as part of a riding model, with C—H = 0.93–0.97 Å, and with U_equiv_ values set at 1.2–1.5 U_equiv_ of the parent atoms.

### Crystal data

**Table:**

C_18_H_15_NO_3_	*F*(000) = 616
*M**_r_* = 293.31	*D*_x_ = 1.302 Mg m^−^^3^
Monoclinic, *P*2_1_/*c*	Mo *K*α radiation, λ = 0.71073 Å
Hall symbol: -P 2ybc	Cell parameters from 1073 reflections
*a* = 10.030 (2) Å	θ = 2.3–19.4°
*b* = 19.223 (3) Å	µ = 0.09 mm^−^^1^
*c* = 7.9652 (17) Å	*T* = 291 K
β = 103.073 (3)°	Block, yellow
*V* = 1495.9 (5) Å^3^	0.24 × 0.20 × 0.18 mm
*Z* = 4	

### Data collection

**Table:**

Bruker SMART APEX CCD diffractometer	2604 independent reflections
Radiation source: sealed tube	1604 reflections with *I* > 2σ(*I*)
graphite	*R*_int_ = 0.057
phi and ω scans	θ_max_ = 25.0°, θ_min_ = 2.1°
Absorption correction: multi-scan (*SADABS*; Bruker, 2000)	*h* = −11→11
*T*_min_ = 0.979, *T*_max_ = 0.984	*k* = −22→20
8910 measured reflections	*l* = −9→9

### Refinement

**Table:**

Refinement on *F*^2^	Secondary atom site location: difference Fourier map
Least-squares matrix: full	Hydrogen site location: inferred from neighbouring sites
*R*[*F*^2^ > 2σ(*F*^2^)] = 0.048	H-atom parameters constrained
*wR*(*F*^2^) = 0.129	*w* = 1/[σ^2^(*F*_o_^2^) + (0.0588*P*)^2^] where *P* = (*F*_o_^2^ + 2*F*_c_^2^)/3
*S* = 1.00	(Δ/σ)_max_ = 0.010
2604 reflections	Δρ_max_ = 0.18 e Å^−^^3^
201 parameters	Δρ_min_ = −0.15 e Å^−^^3^
0 restraints	Extinction correction: *SHELXL97*, Fc^*^=kFc[1+0.001xFc^2^λ^3^/sin(2θ)]^-1/4^
Primary atom site location: structure-invariant direct methods	Extinction coefficient: 0.015 (2)

### Special details

**Table:**

Geometry. All e.s.d.'s (except the e.s.d. in the dihedral angle between two l.s. planes) are estimated using the full covariance matrix. The cell e.s.d.'s are taken into account individually in the estimation of e.s.d.'s in distances, angles and torsion angles; correlations between e.s.d.'s in cell parameters are only used when they are defined by crystal symmetry. An approximate (isotropic) treatment of cell e.s.d.'s is used for estimating e.s.d.'s involving l.s. planes.
Refinement. Refinement of *F*^2^ against ALL reflections. The weighted *R*-factor *wR* and goodness of fit *S* are based on *F*^2^, conventional *R*-factors *R* are based on *F*, with *F* set to zero for negative *F*^2^. The threshold expression of *F*^2^ > σ(*F*^2^) is used only for calculating *R*-factors(gt) *etc*. and is not relevant to the choice of reflections for refinement. *R*-factors based on *F*^2^ are statistically about twice as large as those based on *F*, and *R*- factors based on ALL data will be even larger.Least-squares planes (*x*,*y*,*z* in crystal coordinates) and deviations from them (* indicates atom used to define plane)8.2801 (0.0047) *x* + 9.1394 (0.0112) *y* - 3.8457 (0.0039) *z* = 0.2003 (0.0031)* 0.0099 (0.0018) C1 * 0.0019 (0.0019) C2 * -0.0097 (0.0019) C3 * -0.0087 (0.0017) C4 * 0.0066 (0.0018) C5 * 0.0165 (0.0017) C6 * -0.0108 (0.0017) C7 * -0.0130 (0.0017) C8 * 0.0072 (0.0016) N1Rms deviation of fitted atoms = 0.0101- 0.9358 (0.0101) *x* - 10.1188 (0.0159) *y* + 6.7251 (0.0044) *z* = 4.3984 (0.0037)Angle to previous plane (with approximate e.s.d.) = 54.26 (0.07)* -0.0091 (0.0016) C10 * 0.0153 (0.0017) C11 * -0.0081 (0.0018) C12 * -0.0053 (0.0019) C13 * 0.0114 (0.0018) C14 * -0.0042 (0.0016) C15Rms deviation of fitted atoms = 0.0097

### Fractional atomic coordinates and isotropic or equivalent isotropic displacement parameters (Å^2^)

**Table:**

	*x*	*y*	*z*	*U*_iso_*/*U*_eq_	
C1	0.3367 (2)	0.08044 (12)	0.8614 (3)	0.0535 (7)	
H1	0.4005	0.0494	0.9231	0.064*	
C2	0.3057 (3)	0.13943 (12)	0.9370 (3)	0.0620 (7)	
H2	0.3483	0.1490	1.0509	0.074*	
C3	0.2092 (3)	0.18631 (13)	0.8436 (3)	0.0613 (7)	
H3	0.1880	0.2267	0.8963	0.074*	
C4	0.1464 (2)	0.17291 (11)	0.6764 (3)	0.0501 (6)	
H4	0.0825	0.2041	0.6154	0.060*	
C5	0.1782 (2)	0.11209 (10)	0.5963 (3)	0.0426 (6)	
C6	0.1348 (2)	0.08293 (10)	0.4309 (3)	0.0445 (6)	
C7	0.2020 (2)	0.02004 (11)	0.4333 (3)	0.0477 (6)	
H7	0.1906	−0.0104	0.3403	0.057*	
C8	0.2880 (2)	0.00854 (10)	0.5917 (3)	0.0449 (6)	
C9	0.3887 (2)	−0.04547 (11)	0.6464 (3)	0.0473 (6)	
C10	0.3774 (2)	−0.10994 (10)	0.5398 (3)	0.0411 (6)	
C11	0.4968 (3)	−0.13982 (12)	0.5151 (3)	0.0536 (7)	
H11	0.5808	−0.1192	0.5626	0.064*	
C12	0.4918 (3)	−0.20014 (12)	0.4201 (4)	0.0670 (8)	
H12	0.5722	−0.2191	0.4002	0.080*	
C13	0.3690 (3)	−0.23235 (13)	0.3550 (3)	0.0674 (8)	
H13	0.3662	−0.2732	0.2917	0.081*	
C14	0.2502 (3)	−0.20418 (12)	0.3833 (3)	0.0611 (7)	
H14	0.1671	−0.2265	0.3414	0.073*	
C15	0.2542 (2)	−0.14261 (11)	0.4742 (3)	0.0497 (6)	
H15	0.1734	−0.1231	0.4912	0.060*	
C16	0.0427 (2)	0.11668 (12)	0.2879 (3)	0.0496 (6)	
C17	−0.0756 (3)	0.10378 (13)	−0.0047 (3)	0.0640 (7)	
H17A	−0.0512	0.0856	−0.1073	0.077*	
H17B	−0.0680	0.1541	−0.0066	0.077*	
C18	−0.2186 (3)	0.08400 (16)	−0.0048 (4)	0.0880 (10)	
H18A	−0.2252	0.0343	0.0015	0.132*	
H18B	−0.2785	0.1003	−0.1089	0.132*	
H18C	−0.2446	0.1046	0.0928	0.132*	
N1	0.27358 (18)	0.06663 (9)	0.6934 (2)	0.0438 (5)	
O1	0.48475 (18)	−0.03908 (9)	0.7719 (2)	0.0698 (5)	
O2	0.01794 (18)	0.07657 (8)	0.1471 (2)	0.0672 (5)	
O3	−0.00443 (17)	0.17415 (8)	0.2920 (2)	0.0662 (5)	

### Atomic displacement parameters (Å^2^)

**Table:**

	*U*^11^	*U*^22^	*U*^33^	*U*^12^	*U*^13^	*U*^23^
C1	0.0636 (17)	0.0482 (14)	0.0423 (14)	0.0019 (12)	−0.0016 (12)	−0.0008 (11)
C2	0.080 (2)	0.0534 (15)	0.0490 (16)	0.0019 (14)	0.0082 (14)	−0.0108 (13)
C3	0.0735 (19)	0.0484 (15)	0.0631 (18)	0.0056 (13)	0.0175 (15)	−0.0091 (13)
C4	0.0506 (15)	0.0421 (13)	0.0576 (17)	0.0043 (11)	0.0123 (13)	0.0015 (12)
C5	0.0403 (14)	0.0374 (12)	0.0488 (14)	0.0011 (10)	0.0075 (12)	0.0033 (11)
C6	0.0471 (15)	0.0389 (12)	0.0440 (14)	0.0024 (11)	0.0033 (11)	−0.0009 (11)
C7	0.0532 (16)	0.0408 (13)	0.0445 (15)	−0.0015 (11)	0.0015 (12)	−0.0058 (10)
C8	0.0510 (15)	0.0372 (12)	0.0431 (14)	0.0017 (11)	0.0035 (12)	−0.0031 (10)
C9	0.0464 (15)	0.0434 (13)	0.0485 (15)	0.0003 (11)	0.0028 (13)	0.0015 (11)
C10	0.0458 (15)	0.0342 (12)	0.0406 (13)	0.0014 (11)	0.0040 (11)	0.0066 (10)
C11	0.0483 (16)	0.0473 (14)	0.0643 (17)	0.0002 (12)	0.0109 (13)	0.0001 (12)
C12	0.0695 (19)	0.0521 (16)	0.084 (2)	0.0083 (14)	0.0271 (16)	−0.0052 (15)
C13	0.084 (2)	0.0468 (15)	0.0695 (19)	−0.0001 (16)	0.0127 (17)	−0.0128 (13)
C14	0.0613 (18)	0.0481 (15)	0.0678 (18)	−0.0107 (13)	0.0020 (14)	−0.0026 (13)
C15	0.0471 (16)	0.0444 (13)	0.0550 (16)	0.0025 (11)	0.0061 (13)	0.0017 (12)
C16	0.0505 (16)	0.0441 (14)	0.0505 (16)	0.0011 (12)	0.0037 (12)	0.0040 (12)
C17	0.069 (2)	0.0711 (17)	0.0429 (15)	0.0018 (15)	−0.0064 (14)	0.0072 (13)
C18	0.072 (2)	0.113 (2)	0.074 (2)	−0.0102 (18)	0.0051 (17)	0.0196 (18)
N1	0.0485 (12)	0.0381 (10)	0.0412 (11)	−0.0003 (9)	0.0027 (9)	−0.0013 (8)
O1	0.0645 (12)	0.0669 (12)	0.0635 (12)	0.0164 (9)	−0.0162 (10)	−0.0144 (9)
O2	0.0783 (13)	0.0639 (11)	0.0487 (11)	0.0165 (9)	−0.0083 (9)	−0.0023 (9)
O3	0.0722 (13)	0.0488 (10)	0.0679 (13)	0.0143 (9)	−0.0041 (10)	0.0056 (9)

### Geometric parameters (Å, °)

**Table:**

C1—C2	1.353 (3)	C10—C11	1.382 (3)
C1—N1	1.370 (3)	C11—C12	1.379 (3)
C1—H1	0.9300	C11—H11	0.9300
C2—C3	1.406 (3)	C12—C13	1.372 (3)
C2—H2	0.9300	C12—H12	0.9300
C3—C4	1.363 (3)	C13—C14	1.373 (3)
C3—H3	0.9300	C13—H13	0.9300
C4—C5	1.403 (3)	C14—C15	1.383 (3)
C4—H4	0.9300	C14—H14	0.9300
C5—N1	1.393 (3)	C15—H15	0.9300
C5—C6	1.407 (3)	C16—O3	1.205 (3)
C6—C7	1.382 (3)	C16—O2	1.337 (3)
C6—C16	1.448 (3)	C17—O2	1.450 (3)
C7—C8	1.376 (3)	C17—C18	1.484 (3)
C7—H7	0.9300	C17—H17A	0.9700
C8—N1	1.406 (2)	C17—H17B	0.9700
C8—C9	1.445 (3)	C18—H18A	0.9600
C9—O1	1.228 (3)	C18—H18B	0.9600
C9—C10	1.492 (3)	C18—H18C	0.9600
C10—C15	1.379 (3)		
			
C2—C1—N1	119.7 (2)	C10—C11—H11	119.9
C2—C1—H1	120.2	C13—C12—C11	120.4 (2)
N1—C1—H1	120.2	C13—C12—H12	119.8
C1—C2—C3	120.1 (2)	C11—C12—H12	119.8
C1—C2—H2	119.9	C12—C13—C14	119.9 (2)
C3—C2—H2	119.9	C12—C13—H13	120.1
C4—C3—C2	120.4 (2)	C14—C13—H13	120.1
C4—C3—H3	119.8	C13—C14—C15	120.0 (2)
C2—C3—H3	119.8	C13—C14—H14	120.0
C3—C4—C5	120.0 (2)	C15—C14—H14	120.0
C3—C4—H4	120.0	C10—C15—C14	120.3 (2)
C5—C4—H4	120.0	C10—C15—H15	119.8
N1—C5—C4	117.9 (2)	C14—C15—H15	119.8
N1—C5—C6	107.35 (18)	O3—C16—O2	123.5 (2)
C4—C5—C6	134.7 (2)	O3—C16—C6	125.0 (2)
C7—C6—C5	106.80 (19)	O2—C16—C6	111.5 (2)
C7—C6—C16	128.7 (2)	O2—C17—C18	110.6 (2)
C5—C6—C16	124.45 (19)	O2—C17—H17A	109.5
C8—C7—C6	110.83 (19)	C18—C17—H17A	109.5
C8—C7—H7	124.6	O2—C17—H17B	109.5
C6—C7—H7	124.6	C18—C17—H17B	109.5
C7—C8—N1	106.01 (18)	H17A—C17—H17B	108.1
C7—C8—C9	129.9 (2)	C17—C18—H18A	109.5
N1—C8—C9	123.6 (2)	C17—C18—H18B	109.5
O1—C9—C8	122.7 (2)	H18A—C18—H18B	109.5
O1—C9—C10	119.4 (2)	C17—C18—H18C	109.5
C8—C9—C10	117.9 (2)	H18A—C18—H18C	109.5
C15—C10—C11	119.2 (2)	H18B—C18—H18C	109.5
C15—C10—C9	122.7 (2)	C1—N1—C5	121.86 (18)
C11—C10—C9	118.0 (2)	C1—N1—C8	129.14 (19)
C12—C11—C10	120.2 (2)	C5—N1—C8	109.00 (18)
C12—C11—H11	119.9	C16—O2—C17	116.98 (18)
			
N1—C1—C2—C3	0.0 (4)	C10—C11—C12—C13	−2.4 (4)
C1—C2—C3—C4	−0.2 (4)	C11—C12—C13—C14	0.5 (4)
C2—C3—C4—C5	−0.1 (4)	C12—C13—C14—C15	1.4 (4)
C3—C4—C5—N1	0.6 (3)	C11—C10—C15—C14	−0.6 (3)
C3—C4—C5—C6	−179.7 (2)	C9—C10—C15—C14	−176.8 (2)
N1—C5—C6—C7	1.5 (2)	C13—C14—C15—C10	−1.3 (4)
C4—C5—C6—C7	−178.2 (2)	C7—C6—C16—O3	−174.1 (2)
N1—C5—C6—C16	−175.5 (2)	C5—C6—C16—O3	2.2 (4)
C4—C5—C6—C16	4.8 (4)	C7—C6—C16—O2	4.5 (3)
C5—C6—C7—C8	−1.1 (3)	C5—C6—C16—O2	−179.2 (2)
C16—C6—C7—C8	175.7 (2)	C2—C1—N1—C5	0.5 (3)
C6—C7—C8—N1	0.3 (3)	C2—C1—N1—C8	−178.6 (2)
C6—C7—C8—C9	−172.0 (2)	C4—C5—N1—C1	−0.8 (3)
C7—C8—C9—O1	157.9 (2)	C6—C5—N1—C1	179.45 (18)
N1—C8—C9—O1	−13.2 (4)	C4—C5—N1—C8	178.43 (18)
C7—C8—C9—C10	−19.2 (4)	C6—C5—N1—C8	−1.3 (2)
N1—C8—C9—C10	169.63 (18)	C7—C8—N1—C1	179.8 (2)
O1—C9—C10—C15	139.8 (2)	C9—C8—N1—C1	−7.3 (3)
C8—C9—C10—C15	−43.0 (3)	C7—C8—N1—C5	0.6 (2)
O1—C9—C10—C11	−36.4 (3)	C9—C8—N1—C5	173.6 (2)
C8—C9—C10—C11	140.8 (2)	O3—C16—O2—C17	−2.7 (3)
C15—C10—C11—C12	2.5 (3)	C6—C16—O2—C17	178.65 (19)
C9—C10—C11—C12	178.8 (2)	C18—C17—O2—C16	−89.0 (3)

### Hydrogen-bond geometry (Å, °)

**Table:**

*D*—H···*A*	*D*—H	H···*A*	*D*···*A*	*D*—H···*A*
C1—H1···O1^i^	0.93	2.45	3.163 (3)	134
C14—H14···O3^ii^	0.93	2.58	3.455 (3)	157

Symmetry codes: (i) −*x*+1, −*y*, −*z*+2; (ii) −*x*, *y*−1/2, −*z*+1/2.
